# Incidence of cardiovascular disease in healthy Swedish peripheral blood stem cell donors – a nationwide study

**DOI:** 10.1038/s41409-023-02196-w

**Published:** 2024-01-11

**Authors:** Simon Pahnke, Hans Hägglund, Gunnar Larfors

**Affiliations:** Unit of Haematology, Department of Medical Sciences, Uppsala University, Uppsala, Sweden

**Keywords:** Epidemiology, Stem-cell research

## Abstract

Granulocyte colony-stimulating factor (G-CSF) is used in a majority of healthy donors to obtain peripheral blood stem cells for allogeneic stem cell transplantation. Since high levels of G-CSF activates endothelial cells and can induce a pro-coagulatory state, and fuelled by case reports of cardiovascular events in donors, some concerns have been raised about a potential for an increased risk of cardiovascular events for the donors after donation. We studied the incidence of cardiovascular disease following stem cell donation in a Swedish national register based cohort of 1098 peripheral blood stem cell donors between 1998 and 2016. The primary objective was to evaluate if the incidence of cardiovascular disease was increased for donors treated with G-CSF. The incidence of any new cardiovascular disease was 6.0 cases per 1000 person years, with a median follow up of 9.8 years. The incidence did not exceed that of age- sex- and residency-matched population controls (hazard ratio 0.90, 95% confidence interval (CI) 0.76–1.07, *p*-value 0.23), bone marrow donors, or non-donating siblings. Long-term cardiovascular disease incidence was not increased in this national register based study of peripheral blood stem cell donors treated with G-CSF.

## Introduction

During the last 20 years G-CSF (granulocyte-colony stimulating factor) has been used for blood stem cell mobilisation in healthy blood stem cell donors.

Endogenous G-CSF works as a regulator of myeloid progenitor cell proliferation and maturation into mature neutrophils, mainly mediated through effects on the transmembrane G-CSF receptor. The G-CSF receptor is found on both hematopoietic stem cells and myeloid progenitors, as well as mature cells, such as neutrophilic granulocytes, monocytes and lymphocytes. The receptor is also expressed in a variety of non-hematopoietic cells, including cardiovascular, neuronal, endothelial and placental cells [1, 2].

G-CSF and haematopoietic stem cells have been suggested to play a role in the development of atherosclerosis, and reports of severe cardiovascular adverse events has led to a concern that G-CSF treatment of healthy donors would increase the risk of cardiovascular events [3, 4]. The short-term health risks associated with G-CSF treatment and donation have been well described and are generally considered acceptable [5–8], although Halter et al., in a large retrospective study, reported a higher number of cases (*n* = 7 vs. *n* = 1) of pulmonary embolism and/or deep venous thrombosis in peripheral blood stem cell donors (PBSC) donors than in bone marrow (BM) donors [4].

Less is known about medium to long-term effects on the incidence of cardiovascular disease (CVD).

## Aim

To investigate if the incidence of cardiovascular disease in Swedish peripheral blood stem cell donors is increased after treatment with G-CSF.

## Methods

Combining data from several national Swedish population based registers, we present a prospective register-based cohort study of the incidence of new cardiovascular disease after peripheral blood stem cell donation.

### Data collection

**Fig. 1:**
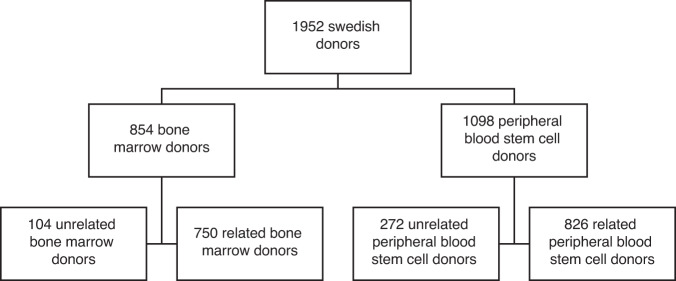
Donors included in study. 1098 peripheral blood stem cell donors and 854 bone marrow donors were identified for the study.

A cohort of related (donating to a sibling, child or parent) haematopoietic stem cell donors between 1977 and 2014 was collected from all Swedish centres for allogeneic stem cell transplantation (Fig. 1). In total, 1576 first time related donors were identified with complete records of personal national identification number, stem cell source, and donation date. For 19 additional donors not all necessary information could not be verified and they were excluded from the study.

An additional 376 unrelated Swedish donors previously included in the Nordic Register for Haematopoietic Stem cell Donors (NRHSD), donating between 1998 and 2014, were also included in the study, making the number of peripheral blood stem cell donors 1098 [8].

A database was created by linking data from Swedish national population based registers; *The Swedish Multi-Generation Register, The Swedish Patient Register, and The Swedish Cause-of-death register*, containing data on all incident diagnoses of cardiovascular disease for the 1098 peripheral blood stem cell donors (PBSC), 1062 siblings and 854 bone marrow donors. For each PBSC donor, five age-, sex- and county of residence-matched controls were assigned from the general Swedish population, 5495 in total. The linkage was performed at the Swedish National Board for Health and Welfare using personal identity numbers, which were then removed before delivery of the datasets for statistical analyses.

### The Swedish Multi-Generation Register

The identity of all the donors’ siblings was obtained from The Multi-Generation Register, Statistics Sweden. The register is derived from Swedish population statistics and include information about the identity of parents, siblings and children for Swedish residents born after 1931 and resident in Sweden at some point in time from 1961 and onwards [9].

### The Swedish Patient Register

The Patient Register includes diagnoses in ICD-format from admissions to hospital since 1965, and specialised out-patient care since 2001 [10]. The patient register was used as the primary source for diagnoses of cardiovascular disease, supplemented with data the Swedish Cause-of-death Register for those without a relevant cardiovascular disease diagnosis before time of death.

### The Swedish Cause-of-death Register

Since 1952, dates and causes of death are recorded in the Causes of Death Register [11].

### Classification of cardiovascular disease

Cases of cardiovascular disease was classified according to ICD-8, ICD-9 or ICD-10 depending on year of diagnosis, Supplementary Appendix 1.

### Statistical analysis

For data analysis, statistical software SAS version 9.4 for Windows (SAS Institute, Cary, NC) was used.

Cardiovascular disease incidences for donors and their comparison groups were modelled by multivariable Cox regression, using the SAS procedure PHREG [12, 13], with age at donation and sex (male/female) included as variables.

To adjust for potential time trends in diagnosis of cardiovascular disease, and because bone marrow donors were more likely to have donated during the use of ICD-code classification ICD-8 and ICD-9, year of donation was also included as a potential confounding factor in the models comparing disease incidence between PBSC and BM donors.

For each disease category, individuals with a diagnosis of the examined disease prior to the time of donation were excluded from that analysis.

Relative risks, compared to population controls, siblings and bone marrow donors, were estimated as hazard ratios with 95% Cis, and the results are presented both as crude number of events, event rates per 1000 person-years of follow up, and in models adjusted for confounding factors.

Mortality for PBSC donors and population controls were calculated using data from the Cause of death register. All-cause mortality and CVD-specific mortality was compared in separate regression models. Two different models were analysed; one with age at donation and sex as possible confounders, and one with the addition of a weighted Charlson Comorbidity Index variable, calculated according to Ludvigsson et al. [14].

### Ethical review

The study was approved by the regional ethical vetting board of Stockholm, 98-259, and Uppsala, 2016-497.

## Results

### Donor characteristics

1576 related and 376 unrelated first time donors were included in the study.

Population based controls for PBSC donors were well matched for sex, age, and highest education level reached, Table 1.

**Table 1:** Study participant characteristics.

Characteristic	PBSC donors	Population controls^a^	Bone marrow donors	Non donating siblings
	*N* (%)	*N* (%)	*N* (%)	*N* (%)
Number	1098	5495	854	1222
Sex				
Male	642 (58)	3202 (58)	449 (53)	629 (51)
Female	456 (42)	2293 (42)	405 (47)	593 (49)
Age at donation, years				
0–18	16 (1)	80 (1)	294 (34)	60 (5)
18–29	160 (15)	799 (15)	165 (19)	134 (11)
30–39	264 (24)	1321 (24)	184 (22)	142 (12)
40–49	296 (27)	1484 (27)	136 (16)	252 (21)
50–59	226 (20)	1131 (21)	61 (7)	365 (30)
60–69	122 (11)	610 (11)	14 (2)	226 (18)
70–79	14 (1)	70 (1)	–	43 (4)
Median	43.8	43.8	27.8	50.9
Range	1.7–76.3	1.7–76.3	0.3–68.9	0–77.5
Relation to recipient				
Related	826 (75)		750 (88)	
Unrelated	272 (25)		104 (12)	
Year of donation				
1970–1979			2 (<1)	
1980–1989			232 (27)	
1990–1999	117 (11)		328 (38)	
2000–2009	701 (64)		201 (24)	
2010–2014	280 (26)		91 (11)	
Highest education level reached (by 2015)^b^				
Primary	53 (6)	299 (7)	38 (6)	55 (6)
Secondary	126 (15)	733 (17)	110 (17)	170 (18)
Post-secondary /non tertiary	399 (48)	1982 (46)	326 (49)	756 (47)
Tertiary/Master/Doctoral	272 (32)	1261 (29)	185 (28)	277 (30)

^a^Matched 1:5 on age, sex and area of residency at time of donation.

^b^Education level data missing for 248 PBSC donors, 1220 population controls, 195 BM donors and 294 siblings.

Choice of donation method was associated with age, with 95% of donors less than 18 years donating bone marrow, while 91% of donors of age above 60, and all above 70 years, donated PBSC.

Bone marrow donation was used exclusively until the last years of the 1990s. A gradual increase in the proportion of PBSC donation is seen in the decades after introduction of G-CSF. The proportion of PBSC donors had increased to around 75% during the last years of the study (2010–2014).

## Donor cardiovascular disease incidence

### Compared with population based controls

Of 1098 PBSC donors, 60 donors (5.5%) had a diagnoses of cardiovascular disease prior to donation, as detailed in Supplementary Appendix 2. Among the remaining 1038 PBSC donors without any cardiovascular disease before donation, a new diagnosis of cardiovascular disease was recorded for 167 donors (16.5%), with a median follow up of 9.2 years (min: 0–max 21.2). The donors event rate of any new cardiovascular disease, 18.1 cases per 1000 person-years, was not different from that of matched controls (event rate 19.2 cases per 1000 person-years, hazard ratio 0.89 (95% CI 0.75–1.06, *p*-value 0.19).

Similarly, there was no risk difference seen between donors and controls for any specific cardiovascular disease category, although a general non-significant trend towards lower risk for donors can be noted, Table 2.

**Table 2:** Events of cardiovascular disease in peripheral blood stem cell donors compared with age-, sex- and residence-matched controls.

New cardiovascular disease after time of donation	PBSC donors			Matched population controls						
	*N* = 1098	Number of events (%)	Events/1000 person-years	*N* = 5495	Number of events	Events/1000 person-years	Hazard ratio	95% Confidence Interval		*P*-value
Any cardiovascular disease	1038	150 (14.5)	18.1	5022	755 (15.0)	19.2	0.89	0.75	1.06	0.19
Any cardiovascular disease, except hypertension	1065	113 (10.6)	11.3	5137	528 (10.3)	11.0	0.97	0.79	1.19	0.78
Hypertension	1067	95 (8.9)	9.4	5298	534 (10.1)	10.8	0.83	0.66	1.03	0.08
Any heart rhythm disease	1085	44 (4.1)	4.2	5408	231 (4.3)	4.5	0.92	0.67	1.27	0.61
Atrial Fibrillation	1091	24 (2.2)	2.3	5448	153 (2.8)	2.9	0.76	0.50	1.17	0.21
Myocardial infarction and/or Ischaemic heart disease	1085	31 (2.9)	2.9	5430	132 (2.5)	2.6	1.07	0.72	1.58	0.74
Myocardial Infarction	1094	19 (1.7)	1.8	5432	90 (1.7)	1.7	0.98	0.59	1.60	0.92
Ischaemic Heart Disease	1085	29 (2.7)	2.8	5358	126 (2.4)	2.4	0.95	0.63	1.42	0.80
Deep venous thrombosis and/or Pulmonary embolism	1098	17 (1.6)	1.6	5436	102 (1.9)	1.9	0.80	0.48	1.34	0.80
Deep venous thrombosis	1098	10 (0.9)	0.9	5447	66 (1.2)	1.3	0.73	0.38	1.42	0.35
Pulmonary embolism	1098	7 (0.6)	0.7	5480	47 (0.9)	0.9	0.72	0.33	1.59	0.42
Cardiac failure	1097	14 (1.3)	1.3	5464	93 (1.7)	1.8	0.73	0.41	1.28	0.26
Cerebrovascular disease	1094	15 (1.4)	1.4	5435	121 (2.2)	2.3	0.59	0.35	1.01	0.06

### Compared with bone marrow donors

The new cardiovascular disease incidence after donation for 1038 PBSC donors was not different from that of 831 bone marrow donors, (hazard ratio 0.90, 95% CI 0.63–1.27, *p*-value 0.90) in a model adjusted for age, sex, year of donation and year of diagnosis, Table 3. Median follow-up time for bone marrow donors was 18.3 years (0.3–38.3 years).

**Table 3:** Events of cardiovascular disease in peripheral blood stem cell donors compared with bone marrow donors.

New cardiovascular disease after time of donation	PBSC donors			BM donors						
	*N* = 1098	Number of events (%)	Events/1000 person-years	*N* = 854	Number of events (%)	Events/1000 person-years	Hazard ratio	95% Confidence Interval		*P*-value
Any cardiovascular disease	1038	150 (14.5)	15.7	841	153 (18.2)	10.0	0.90	0.63	1.27	0.90
Any cardiovascular disease, except hypertension	1065	113 (10.6)	11.3	844	114 (13.5)	7.3	0.94	0.62	1.40	0.75
Hypertension	1067	95 (8.9)	9.4	848	85 (10.0)	5.3	0.82	0.51	1.30	0.39
Any heart rhythm disease	1085	44 (4.1)	4.2	852	53 (6.2)	3.3	0.87	0.47	1.64	0.67
Atrial Fibrillation	1091	24 (2.2)	2.3	854	28 (3.3)	1.7	0.89	0.44	1.79	0.73
Myocardial infarction and/or Ischaemic heart disease	1085	31 (2.9)	2.9	850	28 (3.3)	1.7	1.12	0.49	2.58	0.79
Myocardial Infarction	1094	19 (1.7)	1.8	853	14 (1.6)	0.9	1.00	0.37	3.21	0.88
Ischaemic Heart Disease	1085	29 (2.7)	2.8	850	25 (2.9)	1.5	1.37	0.55	3.47	1.37
Deep venous thrombosis and/or Pulmonary embolism	1098	17 (1.6)	1.6	854	17 (2.0)	1.0	0.85	0.31	2.36	0.76
Deep venous thrombosis	1098	10 (0.9)	0.9	853	13 (1.5)	0.8	0.60	0.18	1.96	0.40
Pulmonary embolism	1098	7 (0.6)	0.7	854	8 (0.9)	0.5	1.71	0.30	9.68	0.55
Cardiac failure	1097	14 (1.3)	1.3	854	15 (1.8)	0.9	3.12	0.71	13.78	0.13
Cerebrovascular disease	1094	15 (1.4)	1.4	853	19 (2.2)	1.2	0.69	0.24	2.05	0.51

### Compared with non-donating siblings

A separate comparison was made between sibling donors and their non-donating siblings. Only sibling PBSC donors with at least one non-transplant-recipient sibling, alive at the time of the donation, were included in the analyses, in total 694 sibling donors and 1222 siblings. Out of 649 donors without any diagnosis of cardiovascular disease at donation, 107 (16.5%) received a new diagnosis of cardiovascular disease during follow up, compared with 209 (19.6%) of 1091 siblings.

The PBSC donors did not have an increased risk for new cardiovascular disease when compared to their non-donating siblings (hazard ratio 0.86, 95% CI 0.68–1.09, *p*-value 0.21), Table 4.

**Table 4:** Events of cardiovascular disease in related peripheral blood stem cell (PBSC) donors compared with their siblings.

New cardiovascular disease after time of donation	PBSC donors			Siblings						
	*N* = 694	Number of events (%)	Events/1000 person-years	*N* = 1222	Number of events	Events/1000 person-years	Hazard ratio	95% Confidence Interval		*P*-value
Any cardiovascular disease	649	107 (16.5)	17.6	1091	209 (19.6)	22.1	0.86	0.68	1.09	0.21
Any cardiovascular disease, except hypertension	669	74 (11.1)	11.6	1115	146 (13.1)	14.7	0.84	0.63	1.11	0.22
Hypertension	670	74 (11.0)	11.5	1168	147 (12.6)	14.5	0.91	0.69	1.20	0.51
Any heart rhythm disease	684	28 (4.1)	4.1	1193	62 (5.2)	5.8	0.79	0.50	1.24	0.30
Atrial Fibrillation	690	18 (2.6)	2.6	1206	41 (3.4)	3.7	0.83	0.47	1.44	0.50
Myocardial infarction and/or Ischaemic heart disease	684	23 (3.3)	3.4	1186	37 (3.1)	3.4	1.16	0.68	1.96	0.59
Myocardial Infarction	694	16 (2.3)	2.3	1222	31 (2.5)	2.8	1.30	0.63	2.67	0.47
Ischaemic Heart Disease	684	21 (3.1)	3.1	1192	37 (3.1)	3.4	1.08	0.63	1.85	0.79
Deep venous thrombosis and/or Pulmonary embolism	694	12 (1.7)	1.7	994	27 (2.3)	2.5	0.76	0.38	1.50	0.42
Deep venous thrombosis	694	8 (1.2)	1.2	1205	18 (1.5)	1.6	0.75	0.32	1.73	0.50
Pulmonary embolism	694	4 (0.6)	0.6	1216	14 (1.1)	1.3	0.49	0.16	1.51	0.21
Cardiac failure	694	10 (1.4)	1.5	1214	25 (2.1)	2.3	0.77	0.37	1.62	0.49
Cerebrovascular disease	690	11 (1.6)	1.6	1197	27 (2.3)	2.5	0.78	0.39	1.59	0.50

## Donor mortality

Mortality from any cause was registered for 32 of 1098 PBSC donors (2.9%) during follow up, compared to 240 of 5495 population controls (4.4%). All-cause mortality was lower for donors than controls (hazard ratio 0.65, 95% CI 0.45–0.94, *p*-value 0.02), in a regression model including sex and age at donation. If a weighted Charlson Comorbidity Index-variable (CCI) was included in the model, as an adjustment for potential different baseline risks of cardiovascular disease between donors and controls, this difference was no longer significant (hazard ratio 0.70, 95% CI 0.49–1.02, *p*-value 0.06).

Similarly, CVD-specific mortality was lower for donors (1.5%) than for controls (2.4%) (hazard ratio 0.59, 95% CI 0.35–0.99, *p*-value 0.04), in the model including sex and age, but no longer significantly so when the CCI variable was included, (hazard ratio 0.65, 95% CI 0.39–1.09, *p*-value 0.10).

There was no donor mortality registered within the first 12 months after donation. The first registered case occurred after 1.8 years, in a related donor aged 61 at time of donation, dying from pancreatic cancer.

Mortality causes for all deceased PBSC donors can be found in Supplementary Appendix 3.

## Discussion

The number of allogeneic transplantations have been increasing steadily over the last 30 years, with an increase in older recipients, and a gradual increase of donations from both sibling and haplo-identical related donors [15, 16]. An expected consequence of this is the increasing use of older donors, presumably with a higher degree of risk factors for cardiovascular disease.

Stem cell donation is generally safe, and associated with low risk of short-term morbidity. However, since the donors are healthy volunteers, all measures must be taken to avoid negative consequences, both at donation and in a longer perspective. Cardiovascular risk has in this setting been high-lighted in international guidelines [17].

In our analyses, the overall risk of cardiovascular disease after PBSC donation reassuringly does not seem to be increased, compared to population controls, bone marrow donors or the donors´ siblings, even among donors followed for a median of almost ten years. There is even an indication that the risk of new cardiovascular disease is slightly lower for PBSC donors compared to age, sex and county-of-residence matched population controls (hazard ratios ranging from 0.72 to 0.98), likely due to unadjusted differences in cardiovascular disease risk factors. The precision of the individual estimates are however fairly low, as seen in Table 2.

The same signal of a possibly lower cardiovascular risk for PBSC donors is not as evident in the comparisons with bone marrow donors or the donors´ siblings. One explanation could be that remaining confounding factors, not accounted for in our models, are differently distributed between comparison groups, combined with a lower statistical power for these analyses due to the smaller study base.

All-cause and CVD-specific mortality in our study seems to be 30–40% reduced compared to age, sex and residency-matched control, even after adjusting for Charlson Comorbidity Index calculated for diagnoses prior to donation. We believe this to be caused by remaining unmeasured confounders not accounted for in this study.

The cardiovascular disease risk after donation have been previously studied using different study designs than in our study. In a large retrospective study by Halter et al., four fatalities from cardiovascular causes occurred within 30 days after donation in PBSC donors, three cardiac arrests and one subarachnoid haemorrhage [4]. The overall number of serious adverse cardiovascular events, also including one case of supraventricular tachycardia, two myocardial infarctions and one subdural haematoma, were low and were judged to be comparable to that of BM donors.

Martino et al. reported that 23% of donors (58/276) were diagnosed with cardiovascular disease during a median follow-up of 7.8 years, similar to the numbers found in our study [18]. The most frequent diagnosis in this study was hypertension in 48 (17%) patients, while 3.6% had other diagnoses, including 4 strokes, 3 acute myocardial infarctions, and single cases of aortic occlusion, pulmonary embolism, and cardiac valvulopathy. The incidence of cardiovascular disease was estimated to be similar to that expected in the general population, although no presentation of the power of this analysis was included, nor any data on loss to follow up.

In a small study by Cavallaro et al. of 95 PBSC donors, with a median follow up of 3.3 years, one stroke and one case of angina was reported. No comparison to expected rates in the population was made [19].

In a much larger study by Pulsipher at al., including 6788 PBSC donors with a fairly short median follow up of 3 years, the incidence of thrombosis, including stroke, did not differ between PBSC and BM donors. However,the analysis did not adjust for age- and sex- differences between donor groups, and did not report any clear definition of what diagnosis were included, nor absolute numbers of events [7].

### Added knowledge from this study

This study provides an estimate of the long-term incidence rate of cardiovascular disease after donation, in a relatively large national cohort of 1098 healthy PBSC donors. The median follow up of 9.8 years for cardiovascular disease in peripheral blood stem cell donors is, according to our knowledge, the longest so far reported. Outcome measures are clearly defined by ICD-codes from validated national registers, with a very low loss to follow up. The incidence rate for cardiovascular disease is compared to that of both matched population controls as well as to that of BM donors and the donors´ siblings, increasing the reliability of the results.

### Limitations to our study

One limitation to this study is a lack of data regarding the distribution of a majority of known risk factors for cardiovascular disease, such as smoking, obesity, physical activity, that may be different between donors and controls. Those accepted for stem cell donation are likely to be healthier and have fewer cardiovascular disease risk factors than the general population, beyond what can be fully adjusted for in our data, possibly leading to a lower sensitivity to detect an eventual risk increase from G-CSF. Another limitation is that only Swedish donors were included in the study, possibly affecting the generalisability of our results. Even though the cardiovascular disease burden in Sweden is fairly similar to that of most Western European, North and South American countries, the cardiovascular disease burden is considerably lower than that seen in many countries [20] performing stem cell transplantations globally.

We could further only study first-time cardiovascular events following donation, due to the nature of the registered data, and not the effect of G-CSF on donors who had records of cardiovascular disease before donation. This latter small group of donors would be of special interest to follow, as this group today might be more likely to be advised against donating due to worries about negative effects of G-CSF. As well, limitations imposed by study size and the nature of available data, did not allow us to investigate the cardiovascular disease risk separately for several specific donor groups of relevance, such as related/unrelated donors, younger/older donors, or those with pre-existing cardiovascular disease, and this should be considered when interpreting our results.

## Conclusion

In this national, register based cohort study of 1098 Swedish PBSC donors followed for a median of 9.8 years, we found no evidence of an increased risk of new cardiovascular disease after donation.

We conclude that concerns about long-term cardiovascular risk, based on today’s knowledge and appropriate donor selection, should not hinder stem cell collection with G-CSF in healthy volunteers.

### Supplementary information

Appendix 1-3

## Data Availability

The data in our study results are not publicly available due to restrictions according to national data protection legislation. Datasets analysed during the current study are available from the corresponding author upon reasonable request.
