# Supplementary material for: Incidence of cardiovascular disease in healthy Swedish peripheral blood stem cell donors – a nationwide study
Source: Bone Marrow Transplant. 2024 Jan 11;59(3):403–8. doi: 10.1038/s41409-023-02196-w (PMC10920189; doi:10.1038/s41409-023-02196-w)
Supplement: Supplementary file 1 — Appendix 1-3 [file 41409_2023_2196_MOESM1_ESM.pdf]

Diagnosis	ICD-10 1997-	ICD -9 1987-1997	ICD-8 1968-1987
Any cardiovascular disease	I00-I82	401-453	400-453
Any cardiovascular disease, except hypertension	I00-I09, I16-I82	406-453	405-453
Hypertension	I10-I15	401-405	400-404
Any heart rhythm disease, including cardiac arrest	I44 –I49	426-427	427.28-427.98
Atrial fibrillation	I48	427D	427.90, 427.92
Myocardial infarction and/or Ischemic heart disease	I21-I22, I24-I25	410-414	410-414
Myocardial infarction	I21-I22	410	410
Ischemic heart disease	I24, I25	411- 414	411-414
Deep venous thrombosis and/or Pulmonary embolism	I26, I80.1, I80.2, I81, I82	415, 325<br>451B, C, W<br>452-453<br>557A	426, 438, 450-453
Deep venous thrombosis	I80.1, I80.2, I81, I82	325<br>451B, C, W<br>452-453<br>557A	438, 451-453
Pulmonary embolism	I26	415	426, 450
Cardiac failure	I50	428	427.0, 428.99
Cerebrovascular disease	I60-I64, G45	430-437	430-438

Appendix 1. Diagnoses of cardiovascular disease with corresponding ICD-10, ICD-9 and ICD-8 codes.

Diagnoses	Number of donors (n =1098)
Hypertension	29
Any arythmia	12
Myocarditis/pericarditis	4
Angina Pectoris	3
Myocardial Infarction	4
Subarachnoid hemorrhage	2
Thrombophlebitis	2
Cerebrovascular disease	2
Heart failure	1
Heart disease, unspecified	1
**Total**	**60**

Appendix 2. Cardiovascular disease before donation in PBSC donors

Main cause of death	Main cause of death, ICD code	Contributing causes of death, ICD codes									
Alzheimer	G309	G309	F009								
Bile duct cancer	C249	C249	Z928	C787							
Bile duct cancer	C249	C249	C799	I10	K567	Z924					
Bile duct cancer	C221	C221	C787	C780							
Brain tumor	C719	C719									
Brain tumor	C719	C719									
Brain tumor	D432	D432									
Cerebrovascular disease	I678	I678	M353								
Cholecystitis	K801	I469	A419	K918	K261	K802	Y839	K819			
Chronic Obstructvie Pulmonary Disease	J440	C260									
Colon cancer	C189	C189	C798								
Diabetes Mellitus	E142	N19	N189	E149	A410						
Endometrial cancer	C549	I269	C549	R18	Z929						
Heart failure	I509	I469	I519	I509							
Intoxication	X42	T404	X42	T407							
Ischemic heart disease	I259	J189	I5099	I259	J449						
Kidney failure	N19	R688	E872	N19	I5091	I48	E149	E669			
Liver cancer	C220	R579	C220	I709	I517	I251	I258	J81	K746	D383	D377
Liver cancer	C220	C220	C780								
Liver/bile duct cancer	D376	R579	R5809	D376	K762	C911					
Lung cancer	C349	C349									
Lungcancer	C349	C349	I48								
Multiple myeloma	C900	C900									
Myelofibrosis	D474	D474									
Myocardial infarction	I219	I2199	I251	I2199							
Myocardial infarction	I219	I509	I219	I709	C23						
Myocardial infarction	I219	I509	I219	I48	I10	F03					
Pancreatic cancer	C259	C259									
Pancreatic cancer	C259	C787	C799	C259	C795	C80					
Prostate cancer	C61	G931	R579	R688	C61	C798					
Sepsis	A419	A419	E149								
Unknown	R999										

Appendix 3. Mortality causes of PBSC donors
